# Prevalence and factors associated with condom use among women aged 15-49 years in Rwanda using a survey logistic regression model: evidence from the 2019/20 Rwanda Demographic and Health Survey

**DOI:** 10.11604/pamj.2023.46.121.41640

**Published:** 2023-12-28

**Authors:** Mkhombiseni Zamani Sithole, Jesca Mercy Batidzirai, Ashenafi Argaw Yirga, Alfred Musekiwa

**Affiliations:** 1 School of Mathematics, Statistics and Computer Science, College of Agriculture, Engineering and Science, University of KwaZulu-Natal, Pietermaritzburg, South Africa,; 2 School of Nursing and Public Health, College of Health Sciences, University of KwaZulu-Natal, Durban, South Africa,; 3 School of Health Systems and Public Health, Faculty of Health Sciences, University of Pretoria, Pretoria, South Africa

**Keywords:** Condom use, reproductive women, HIV prevention

## Abstract

**Introduction:**

although Rwanda's HIV prevalence has declined, many people are still acquiring or living with it. Among other methods of HIV prevention, condoms are a safe and reliable method in addition to preventing pregnancy and other sexually transmitted infections, especially when used properly. This study aimed to determine the prevalence and determinants of condom use during last sexual intercourse among reproductive-aged women in Rwanda.

**Methods:**

using data from the cross-sectional, nationally representative Rwanda Demographic and Health Survey (RDHS) conducted in 2019/2020, we carried out secondary data analysis. A multivariable logistic regression model was applied to determine the factors associated with condom use. All analyses from the model were adjusted for unequal sampling probabilities using survey weights.

**Results:**

results showed a 10.8% prevalence of condom use. The odds of condom use during last sex were significantly lower for women who lived with a man (adjusted odds ratio [aOR]= 0.10, CI=0.08: 0.13) and those from the Southern region (aOR=0.69, CI= 0.52 to 0.92) but were significantly higher for those with primary education (aOR=1.38, CI= 1.00: 1.88). Also, the rich were more significantly associated with condom use compared to the poor (aOR=1.53, 95% CI= 1.20: 1.93). Those who had three or more sexual partners had higher odds of condom use than those with one partner (aOR=3.12, CI= 2.50: 3.89).

**Conclusion:**

based on the results, health promotion interventions aimed at raising awareness on HIV prevention should, therefore, target the groups that were found to have a high risk of not using condoms.

## Introduction

Human Immunodeficiency Virus/Acquired Immune Deficiency Syndrome (HIV/AIDS) remains one of the leading causes of morbidity and mortality throughout the world [1-3]. According to the most recent studies, there were roughly 38.0 million HIV-positive people globally in 2019 [4]. Approximately 25.4 million (67%) of them were receiving antiretroviral therapy (ART). There were 1.7 million new HIV infections and 690,000 people dying from AIDS-related illnesses [4,5]. Despite making up only 12% of the world's population, Sub-Saharan Africa disproportionately bears the burden of HIV infection, contributing more than 68% of the infection rate worldwide [6]. In Rwanda, HIV incidence peaked in the mid-1990s and appeared to fall after the deployment of population preventative strategies such as blood screening, health education, and prevention services such as condom distribution [7,8]. According to the Rwanda Demographic and Health Surveys (RDHS), the national HIV prevalence rate among adults in Rwanda remained stable at 3% from 2005 to 2020 and is on track to achieve the UNAIDS 95-95-95 target by 2030 (95% of persons with HIV will be aware of their status, 95% of those diagnosed with HIV infection will receive ART, and 95% of people using ART will have successfully suppressed the virus). Rwanda, like any other country, needs to review current programs with the intention to identify potential factors that may hinder the realization of these goals. Hence, the need for this current study. HIV prevalence among adult Rwandese women and men in 2020 was 3.7% and 2.2%, respectively [9]. In addition, young women have been found to bear a disproportionate share of the HIV epidemic in Sub-Saharan Africa than men [10]. Women aged 15-49 years had the second highest HIV prevalence after women aged 50 years and above in 2020 [11]. Heterosexual transmission through unprotected sex is the predominant cause of HIV infection in Rwanda, with no statistics on the prevalence of HIV among homosexuals [12]. Some HIV prevention measures include condom use, male circumcision, and Pre-Exposure Prophylaxis (PrEP), which reduces the risk of acquiring HIV among people at risk of getting HIV [13]. Condoms, on the other hand, are the most easily and commonly available with an added advantage of preventing various sexually transmitted infections (STIs) and unwanted pregnancies, making them one of the most flexible and cost-effective health commodities. Based on evidence from longitudinal studies, the effectiveness of condoms to prevent HIV is estimated to be 80-85%, although it may be as high as 95% when used consistently and correctly [14].The UNAIDS 2016-2021 strategy stressed the importance of condom usage, calling for the availability of 20 billion condoms and setting a target of 90% condom use during sex with a non-regular partner by 2020 [15].

Results from population-based surveys for countries that report a higher rate of condom use have consistently showed lower HIV prevalence than countries which reported lower usage of condoms [10,16,17]. Previous studies highlighted that male condom use was a common practice among young women in Sub-Saharan Africa, compared to female condom use [10]. Rwanda national condom policy prioritizes its focus on vulnerable groups who are at risk of acquiring HIV. These include young women who may have poor negotiating skills during sex and have a higher chance of infection than males, in addition to them being the ones who suffer pregnancy consequences. Furthermore, their rights of accessing appropriate reproductive health care products such as condoms distribution might be limited due to social and cultural norms which consider adolescents engaging in premarital sex as immoral [18]. Previous studies across the world showed that level of education, employment status, multiple sexual partners, region, age, living with a partner, and marital status were significantly associated with condom use [10,19,20]. Although findings from these studies may still be valid and relevant, Rwandan population dynamics and other health indicators may have changed in recent times. Therefore, using an up-to-date data set, this study aims to determine factors associated with condom use among women aged 15 to 49 years in Rwanda.

## Methods

**Study Design and setting**: a secondary data analysis of the RDHS dataset from 2019/20 was performed. These surveys employed cross-sectional research designs. The RDHS is implemented in all regions of Rwanda, including rural and urban areas. The DHS is a nationally representative survey that collects information on population health, HIV/AIDS, malaria, and nutrition in each country through a multistage and stratified methodology.

**Study population, and inclusion and exclusion criteria**: the study population consists of sexually active men and women aged 15-49 years who lived in Rwanda at the time of the 2019/2020 RDHS. Participants who were not sexually active and those who never had sex in the past 12 months were excluded.

**Sampling**: the 2012 Rwandan population census was utilized as the sampling frame for the survey, which employed stratified, two-stage cluster random sampling of homes. 14,634 participants who were members of the chosen households and gave their consent to participate made up the household sample size for the 2019-2020 survey. Of these women who were interviewed for the 2019/2020 DHS, 8,481 were eligible to participate in this study as they were aged 15-49 years and sexually active. Since there were no missing values, none of the eligible participants were excluded for reasons of missing data. The 2019-2020 Rwanda DHS report contains additional information on sampling [21].

**Outcome measure**: the primary outcome measure of interest in this study was whether one had used condom during last sex in the past year.

**Measurements**: the participant´s individual recode file was used in a secondary data analysis of the Rwanda DHS 2019/20 available datasets on a number of variables to assess the factors related to condom use during last sex. The outcome variable was condom use during last sex (yes or no). The potential factors associated with condom use during last sex include age category (15-24 years, 25-34 years, 35-49 years), place of residence (rural or urban), region (Kigali, South, West, North, East), marital status, which has been reclassified as living with a partner (Yes, No), highest level of education (no education, primary, secondary, and higher than secondary), employment in the last 12 months (Employed, not employed), wealth quintile/index (poor, middle, rich) and religion (Catholic, Protestant, Adventist, Muslim, and other religions inclusive of traditional, Jehovah witness and no religion).

HIV knowledge variables include ever tested for HIV (Yes, No) and comprehensive knowledge about HIV (yes or no). Comprehensive HIV knowledge included being aware of the possibility of having HIV in a seemingly healthy person, the need for condom use during sexual activity, would be afraid to get HIV from contact with saliva from an infected person, the fact that HIV cannot be contracted through mosquito bites, and the fact that it cannot be acquired through the use of witchcraft or other supernatural means. Participants were questioned regarding their current contraceptive method used (not using contraceptive, pills and injection, implant). HIV discriminatory attitude was measured as “No” to the following questions: people hesitate to take HIV tests because of the reaction of other people if they are positive; people would be ashamed if someone in the family had HIV. The responses to the following questions were likewise measured as “Yes”: would purchase veggies from an HIV-positive seller; should children with HIV be permitted to attend school with children without HIV.

One, two, and three or more imputed numbers were used to recode the lifetime total of sexual partners. Participants were also questioned about whether they had experienced STIs or STI symptoms in the previous 12 months (yes, no, or don't know), if they had ever experienced any kind of gender-based violence (GBV) or experienced any sexual violence. Saying “Yes” to any one or more of the following actions was considered to constitute GBV: being pushed, slapped, struck with a fist or hit with something, kicked, burned, threatened with or attacked with a knife, twisted, or physically coerced into engaging in sexual activity, physically forced to have any other sexual act, or performing any sexual act they did not want. The frequency of reading newspapers, watching TV, and listening to the radio were used to generate the exposure to media variable. The responses “not at all” and “less than once a week and at least once a week” to at least one media source was coded as 0 and 1, respectively. The same applied to watching TV, listening to the radio, and reading the newspaper. The final media exposure variable was coded as 0 for all respondents who indicated “not at all” for any media sources and 1 for respondents who indicated “access to at least newspaper, TV, or radio.”

**Data collection and analysis:** the DHS Program website provided access to the data set [22]. Exploratory data analysis was done to summarize participants´ characteristics, and categorical variables were expressed as frequencies and percentages. Logistic regression models were employed to determine the relationships between condom use during last sex and independent variables, while taking into consideration the complex nature of the survey data. Age in years, residence, region, living with a partner, education, employment, wealth index, religion, having comprehensive knowledge of HIV, current contraceptive method used, ever tested for HIV, having discriminatory attitude, lifetime number of sexual partners, having STI in last 12 months, ever experiencing sexual violence, and having access to media, were the independent factors. Chi-square test was used to assess the significance of variables. We performed a univariate analysis of each predictor variable against the outcome variable, condom use during last sex, and a manual forward stepwise procedure was used to enter all factors with a p - value < 0.2 into the multivariable logistic regression model. Our findings were displayed as crude odds ratios (OR) and adjusted odds ratios (aOR), together with the corresponding 95% confidence intervals (CI) and p-values. The analyses were adjusted for survey weights, and a statistically significant result was one with a p-value less than 0. 05. To account for differential sampling probabilities due to the complicated survey design, all statistical analyses were performed using STATA version 17 with “svy” command. We also used the “svyset” option of “single unit (certainty)” to prevent the model from failing to converge where the numbers were small.

**Institutional review board statement**: the study used secondary data, hence no need for ethical approval. It was done according to DHS guideline. More details on ethical approval for DHS datasets may be accessed from [22].

## Results

**Exploratory data analysis**: Table 1 displays the exploratory data analysis based on the participants´ characteristics were the prevalence of condom use in the past 12 months was 10.8% (914 participants used condom and 7567 did not). Out of the 8,481 eligible women, the majority were aged 35-49 years (44.5), 81.0% women were residing in rural areas, and most participants were from the Eastern region of Rwanda (27.4%). Almost ninety percent (89.9%) of the women were living with a man and 4.8% completed higher level of education. Most participants were currently employed (83.6%) and 41.9% from rich households. Regarding religion of the participants, Catholic religion had most participants (36.8%). Fifty-seven point seven percent (57.7%) of the women had comprehensive HIV knowledge, 95.4% had ever tested for HIV before. Most women did not have HIV discriminatory attitudes (50.8%). With regards to sexual behavior, most participants had one sexual partners (65.0%). Less than 5% reported having had an STI in the previous year (4.9%), reported experiencing sexual violence (2.3%). In terms of access to media, only 3.3% had access to at least one of newspaper, TV or radio. With regards to condom use during last sex with most recent partner, few participants used condoms in their last intercourse (7.3% of female and 4.9% of male). Most women did not use condom during last sex (60.7%). The variables religion and number of sexual partners had 89 and 11 missing values on the data, respectively. A complete case analysis was performed to handle this.

**Table 1:** characteristics of sexually active women aged 15-49 years, interviewed in the 2019/20 Rwanda DHS (N=8,481)

Covariate	Category	Condom use N (%)		Total		p-value
		No	Yes	N	%	
**Age group**	15-24	1093(13.2)	297 (3.3)	1390	16.5	<0.001
	25-34	2964(35.1)	312(3.9)	3276	38.9	
	35-49	3510(41.0)	305(3.6)	3815	44.5	
**Residence**	Urban	1612(15.5)	330(3.5)	1942	19.0	<0.001
	Rural	5955(73.7)	584(7.3)	6539	81.0	
	Kigali	864(12.0)	212(2.8)	1076	14.7	<0.001
**Region**	South	1851(19.1)	186(1.9)	2037	21.0	
	West	1707(19.1)	195(2.2)	1902	21.3	
	North	1251(14.2)	113(1.3)	1364	15.5	
	East	1894(24.8)	208(2.6)	2102	27.4	
**Living with a man**	Yes	7102(83.9)	491(6.0)	7593	89.9	<0.001
	No	465(5.3)	423(4.8)	888	10.1	
**Education**	No education	946(11.3)	57(0.7	1003	12.0	<0.001
	Primary	4923(58.0)	540(6.3)	5463	64.3	
	Secondary	1316(15.8)	258(3.1)	1574	18.9	
	Higher	382(4.1)	59(0.7)	441	4.8	
**Employment**	No	1228(14.3)	182(2.1)	1410	16.4	0.005
	Yes	6339(74.9)	732(8.7)	7071	83.6	
**Wealth index**	Poor	3057(35.3)	277(3.2)	3334	38.5	0.000
	Middle	1500(18.1	121(1.5)	1621	19.6	
	Rich	3010(35.8)	516(6.1)	3526	41.9	
**Religion**	Catholic	2791(32.4)	370(4.3)	3161	36.8	<0.001
	Protestant	3543(43.2)	369(4.5)	3912	47.7	
	Adventist	870(11.5)	120(1.4)	1090	12.9	
	Muslim	137(1.5)	37(0.4)	174	1.9	
	Other religions	50(0.7)	5(0.1)	55	0.7	
**Have comprehensive knowledge of HIV**	No	3213(37.8)	87(4.5)	3600	42.3	0.871
	Yes	4354(51.5)	527(6.2)	4881	57.7	
**Ever tested for HIV**	No	343(3.9)	68(0.7)	411	4.6	<0.001
	Yes	7224(85.4)	846(10.0)	8070	95.4	
**HIV discriminatory attitudes**	No	3858(45.8)	427(5.0)	4285	50.8	0.025
	Yes	3709(43.5)	487(5.7)	4196	49.2	
**Lifetime number of sexual partners**	One	5140(60.6)	376(4.4)	5516	65.0	<0.001
	Two	1614(19.1)	236(2.7)	1850	21.9	
	Three or more	805(9.5)	299(3.6)	1104	13.1	
**Had STI in last 12 Months**	No	7212(85.1)	845(9.9)	8057	95.1	0.001
	Yes	351(4.0)	68(0.8)	419	4.9	
	Do not know	4(0.1)	1(0.0)	5	0.1	
**Pregnancy**	No	547(6.6)	229(2.5)	776	9.1	<0.001
	Yes	7020(82.7)	685(8.2)	7705	90.9	
**Experienced any sexual violence**	No	7390(87.1)	899(10.6)	8289	97.7	0.210
	Yes	177(2.1)	15(0.2)	192	2.3	
**Access to medpercentagesia**	No	7313(86.5)	861(10.2)	8174	96.7	0.003
	Yes	254(2.7)	53(0.6)	307	3.3	

* Note that the percentage presented are weighted. %: percent; HIV: Human Immunodeficiency Virus; CI: confidence interval; GBV: gender based violence

**Results from the models**: results show that 10.8% (95% CI 10.0 to 11.6) of the women used condom during their last sexual intercourse. Table 2 shows results from the univariate and multivariable logistic regression models.

**Table 2:** factors associated with condom use during last sex among sexually active women aged 15-49 years, interviewed in the 2019/20 Rwanda DHS (N=8,841)

	Univariate			Multivariate		
**Characteristics**	**OR**	**95% CI**	**p-Value**	**aOR**	**95% CI**	**p-Value**
**Age in years**						
15-24	Ref					
25-34	0.43	0.35 to 0.54	0.000	1.06	0.80 to 1.42	0.677
35-49	0.34	0.28 to 0.42	0.000	1.26	0.95 to 1.66	0.099
**Residence**						
Urban	Ref					
Rural	0.44	0.37 to 0.53	0.000	0.94	0.74 to 1.19	0.613
**Region**						
Kigali	Ref					
South	0.44	0.35 to 0.55	0.000	0.69	0.52 to 0.92	0.011*
West	0.49	0.39 to 0.62	0.000	0.93	0.71 to 1.23	0.623
North	0.38	0.29 to 0.51	0.000	0.71	0.52 to 0.97	0.032*
East	0.45	0.35 to 0.57	0.000	0.73	0.54 to 0.98	0.035*
**Living with a man**						
No	Ref					
Yes	0.08	0.06 to 0.09	0.000	0.10	0.08 to 0.13	0.000*
**Education**						
No education	Ref					
Primary	1.77	1.32 to 2.37	0.000	1.38	1.00 to 1.88	0.045*
Secondary	3.16	2.28 to 4.38	0.000	1.39	0.95 to 2.04	0.084
Higher	2.59	1.69 to 3.98	0.000	1.16	0.68 to 1.99	0.575
**Employment**						
No	Ref					
Yes	0.81	0.67 to 0.97	0.025	1.10	0.87 to 1.38	0.431
**Wealth index**						
Poor	Ref					
Middle	0.86	0.71 to 1.10	0.282	0.87	0.68 to 1.10	0.237
Rich	1.90	1.59 to 2.24	0.000	1.53	1.20 to 1.93	0.001*
**Religion**						
Catholic	Ref					
Protista	0.78	0.66 to 0.93	0.005	0.86	0.70 to 1.05	0.133
Adventist	0.93	0.74 to 1.16	0.514	1.10	0.85 to 1.43	0.448
Muslim	1.98	1.32 to 2.97	0.001	1.49	0.95 to 2.35	0.081
Other religion	0.66	0.23 to 1.83	0.424	0.70	0.23 to 2.16	0.538
**Have comprehensive Knowledge about HIV**						
No	Ref					
Yes	1.01	0.87 to 1.18	0.879			
**Ever tested for HIV**						
No	Ref					
Yes	0.61	0.46 to 0.80	0.000	1.078	0.77 to 1.48	0.637
**HIV discriminatory attitudes**						
No	Ref					
Yes	1.19	1.01 to 1.40	0.029	0.98	0.83 to 1.18	0.828
**Lifetime number of sex partners**						
1	Ref					
2	1.98	1.65 to 2.39	0.000	1.58	1.28 to 1.96	0.000*
3 or more	5.31	7.37 to 6.45	0.000	3.12	2.50 to 3.89	0.000*
**Had an STI in last 12 months**						
No	Ref					
Yes	1.71	1.28 to 2.28	0.000	1.27	0.91 to 1.78	0.163
Don't know	2.04	0.12 to 0.13	0.533	0.42	0.44 to 4.22	0.462
**Pregnancy**						
No	Ref					
Yes	0.26	0.21 to 0.31	0.000	0.89	0.68 to 1.17	0.391
**Ever experience any sexual violence**						
No	Ref					
Yes	1.29	0.73 to 2.20	0.383			
**Accesses to media**						
No	Ref					
Yes	1.92	1.35 to 2.75	0.000	1.10	0.65 to 1.86	0.729

**Socio-demographic factors**: increasing age was associated with increasing odds of condom use [(OR 0.43, 95% CI= 0.35 to 0.54, < 0.001) and (OR 0.34'> p < 0.001) and (OR 0.34, 95% CI= 0.28 to 0.42,0.001)'> p < 0.001),respectively] of condom use than those aged 15-24 years. However, after adjusting for potential confounders, this was no longer significant. Women who were living with a man had lower odds of condom use than those not living with a man (OR 0.08, 95% CI= 0.06 to 0.09, p < 0.001). This remained significant after taking into account potential confounders (aOR=0.10, 95% CI= 0.80 to 0.13, p < 0.001). Women from Southern region had significantly lower odds (0.44, 95% CI= 0.35 to 0.55, p < 0.001) of using condom in last sex than those coming from Kigali region. After adjusting for potential confounders, this remained significant (aOR=0.69, 95% CI= 0.52 to 0.92, p = 0.011). Women from Protista and other religions had lower but not significant odds [(OR 0.78, 95% CI= 0.66 to 0.93, p =0.005) and (OR 0.66, 95% CI= 0.23 to 1.83, p = 0.424), respectively] of condom use during their last sexual encounter than those from the Catholic religion. All religion categories were no longer significant after adjusting for potential confounders (Table 2). In comparison to women from poor households, those from rich households had significantly higher odds (OR 1.90, 95% CI= 1.59 to 2.24, p < 0.0001). This remained significant after adjusting for potential confounders (aOR=1.53, 95% CI= 1.20 to 1.93, p < 0.001). Women with primary education had higher odds (OR 1.77, 95% CI= 1.32 to 2.37, p <0.001) of condom use than those with no education. This was no longer significant after adjusting for potential confounders. The odds of condom use during their last sexual encounter were lower among those who had been pregnant in the past 24 months than those who were not pregnant (OR 0.26, 95% CI= 0.21 to 0.31, p < 0.001), this was no longer significant after adjusting for potential confounders (Table 2).

**Risky sexual behavior**: the odds of condom use significantly increased with the increasing number of sexual partners [ (OR 1.98, 95 %, CI= 1.65 to 2.39, < 0.001) for subjects with 2 sexual partners'> p < 0.001) for subjects with 2 sexual partners, and (OR 5.31, 95% CI= 7.37 to 6.45, < 0.001) for subjects with 3 or more sexual partners'> p < 0.001) for subjects with 3 or more sexual partners]. This remained significant after adjusting for potential confounders [ (aOR=1.58, 95%CI= 1.28 to 1.96, < 0.001) for subjects with 2 sexual partners'> p < 0.001) for subjects with 2 sexual partners, and (aOR=3.12, CI= 2.50 to 3.89, < 0.001) for subjects with 3 or more sexual partners p < 0.001) for subjects with 3 or more sexual partners] (Table 2).

## Discussion

**Key results and interpretation**: using data from the 2019/2020 Demographic and Health Survey (DHS), the objective of this study was to determine the prevalence and determinants of condom use among sexually active women (15 - 49 years) in Rwanda. To do this, we analyzed data from 8,841 sexually active women, which revealed a prevalence of condom use during last sex among Rwandese women of 10.8%. This is less than 22.2% of women of reproductive age reported in districts of Tanzania [23]. Findings from the adjusted model shows that being in the South, North and East regions, living with a man, primary education, being rich, and having two or more sexual partners, were significantly associated with condom use during last sex. Results also showed that women who lived with a man were less likely to use condoms. This agrees with previous research [10] who found that young women were more likely to use a condom at last sex if they are living separately from their regular partners.

As the wealth index increased, the likelihood of condom uses also increased. This study provided evidence to support the notion that women from low-income families are less likely to use condoms during sexual activity. Similar findings were made by Soler *at al*., 2000 [24] who found that women who reported no financial decision-making responsibilities were less likely to use condoms on a regular basis. This could be attributed to lack of decision-making power Educated women were more likely to use condom than those who never registered for education although only the results for primary level were significant and this is in agreement with a study from Ethiopia [25], which found that educated women were more likely to use contraceptive than their counterparts. Higher levels of education influence attitudes toward condom use, reinforcing the importance of education in creating healthier behaviors for disease prevention [26]. Education plays an imperative role on societal transformation and that education enhances women´s self-esteem, self-confidence, ability to make decisions and freedom of expression [27].

As the number of sexual partners increased, the likelihood of using condom also significantly increased. This is in line with other previously published studies from Eswatini and Japan [28,29]. This result proves previous research that suggested casual sexual interactions were linked to higher odds and more consistent condom usage because many men and women in these types of relationships are unaware of their partners' sexual histories, especially those who take sexual risks [30]. Lastly, women from Kigali were more likely to use condom than women from the other regions of Rwanda. This could be explained by the higher prevalence of HIV in Kigali compared to other regions as found in the 2010 Rwanda Demographic and Health Survey (RDHS) [31]. It is possible that people who live in high HIV prevalence areas turns to be more likely to use condoms in preventing HIV compared to areas with lower HIV prevalence.

### Limitations and generalizability

A few limitations apply to this study. For instance, we cannot rule out recall bias as some participants may have forgotten answers to some of the questions. It is possible that it could have been selection bias due to the complex sampling methodology used in the DHS. We cannot also conclude a causal association between the risk factors and condom use during last sex because this is a secondary data analysis of a cross-sectional study. The original RDHS database included both men and women but this study only focused on women because they are the more vulnerable to consequences like pregnancy and they are at a higher risk of HIV infection than their male counter parts. Therefore, future studies may also include men to investigate their behavior and attitudes toward condom use as well. In addition, we only considered women in the 15-49 age group because they are more likely to acquire HIV infection than men in the same age group [10,11]. Results of this study may be generalized to the whole country of Rwanda as the sample was a good representative of reproductive women in Rwanda.

## Conclusion

In conclusion, we found that condom use among women in Rwanda was extremely low, thus suggesting a need for more educational programs to promote condom use. Living with a man, primary education, being rich, multiple sexual partnership, and residing in the South, North, and East regions were significantly associated with condom use. The Rwandan government may target these groups for more programs on condom use promotion in an effort to reduce HIV transmission.

### What is known about this topic

- *HIV prevalence and condom use: it is well known that HIV prevalence is a major public health concern in Rwanda and condom use is an important strategy for preventing HIV transmission; previous research may have established the significance of condom use in HIV prevention;*
- *Previous research may have suggested that socioeconomic factors such as education and wealth status can influence condom use; individuals with a higher level of education and a higher socioeconomic status, for example, may be more likely to use condoms as a form of protection*.

### What this study adds

- *This study incorporates recent data on condom use among reproductive-aged women from the 2019/20 Rwanda Demographic and Health Survey (RDHS); it provides an up-to-date understanding of condom usage patterns (trends) in Rwanda by analyzing this specific dataset taking into account a nature of a survey design;*
- *Condom use factors: the study identifies specific factors associated with condom use during the most recent sexual intercourse, such as living arrangements, geographic region, education level, wealth status, and the number of sexual partners; this adds to our understanding of the complex factors that influence condom use in Rwanda;*
- *The study concludes by recommending that health promotion interventions take individual-level factors influencing condom use into account; it emphasizes the importance of dealing with relationship dynamics as well as promoting empowerment and self-efficacy as strategies for increasing condom use and HIV prevention*.
